# How Can the Epidemic Curve of COVID-19 in Iran Be Interpreted?

**DOI:** 10.34172/jrhs.2020.27

**Published:** 2020-10-04

**Authors:** Amin Doosti-Irani, Ali Akbar Haghdoost, Farid Najafi, Sana Eybpoosh, Ghobad Moradi, Fahimeh Bagheri Amiri, Leila Mounesan, Ehsan Mostafavi

**Affiliations:** ^1^Department of Epidemiology, School of Public Health, Hamadan University of Medical Sciences, Hamadan, Iran; ^2^Research Center for Health Sciences, Hamadan University of Medical Sciences, Hamadan, Iran; ^3^Modeling in Health Research Center, Institute for Futures Studies in Health, Kerman University of Medical Sciences, Kerman, Iran; ^4^Research Center for Environmental Determinants of Health (RCEDH), Health Institute, Kermanshah University of Medical Sciences, Kermanshah, Iran; ^5^Department of Epidemiology and Biostatistics, Research Centre for Emerging and Reemerging Infectious Diseases, Pasteur Institute of Iran, Tehran, Iran; ^6^Social Determinants of Health Research Center, Research Institute for Health Development, Kurdistan University of Medical Sciences, Sanandaj, Iran

**Keywords:** COVID-19, Epidemics, Iran

## Abstract

**Background:** Iran is one of the countries most affected by COVID-19. This review provides possible interpretations of the observed trend of COVID-19 in Iran.

**Study design:** A rapid review

**Methods:** We reviewed the daily new cases of COVID-19 based on hospitalized and outpatients, reported deaths, and diagnostic testing in Iran.

**Results:** Iran reported its first peak in the number of cases in late March, 2020. From the 1 April to 3 May 2020, the downward trend in the number of cases was started. The death trend also showed a peak in early April as well as a downward trend in late April. During May, the number of death cases showed a stable trend with a daily number of deaths ranging between 50 and 75 cases. Then the number of deaths gradually increased.

**Conclusion:** The epidemic curve in Iran is a function of different factors such number of total tests, change in mitigation policies, and heterogeneities among different provinces in the country. Therefore it should be interpreted under the light of the effect of such factors.

## Introduction

Iran is one of the countries most affected by COVID-19. The first cases of COVID-19 in Iran were detected on 19 February 2020 ^1^.

The shape of the epidemic curve differs from localities worldwide. Based on the epidemic situation, countries can be categorized into different groups: the first group has had the first peak of the VOVID-19, the second group is in the middle that cases are increasing the peak, and the third category is exposing the beginning of rising cases in next wave ^2^. At the same time, some countries may have finished the first wave and are now arrived at the plateau, or some of them have not experienced a serious peak from the beginning and have had sporadic cases. Up to 27 June 2020, countries such as the United State, Brazil, and India have not yet experienced a descending trend; countries like Spain, Italy, United Kingdom, and Germany have reached a steady trend after a declining epidemic curve, and countries such as Iran, Saudi Arabia, Kuwait, and Romania have experienced a bimodal pattern ^1^. The epidemic curve as a visual representation of the epidemic is a useful tool for assessing the time distribution and type of the epidemic. The correct interpretation of the epidemic curve can be useful for better decision making and managing the epidemic.

In this review, we aimed to provide possible interpretations of the observed time trend of COVID-19 in Iran.

## Methods

We reviewed the daily new cases of COVID-19 based on hospitalized and outpatients, reported deaths, and diagnostic testing in Iran up to 28 Jun 2020. Besides, we used the daily reported cases in other countries ^1^.

A confirmed case of COVID-19 is a person with PCR laboratory confirmation of infection, regardless of the clinical signs and symptoms ^3^. The case finding of COVID-19 in Iran is based on both passive case finding among people referring to the hospitals and comprehensive health service centers, and active case finding via the contact tracing of confirmed cases.

## Results

Up to 28 June 2020, 220,669 confirmed cases and 10,508 deaths are reported by Iran’s surveillance system. From early May, the number of reported confirmed COVID-19 cases in Iran has increased again, providing a bimodal epidemic curve. Iran reported its first peak in the number of cases in late March (n=3111). From the 1 April to 3 May 2020, the downward trend in the number of cases was started.

**Figure 1:**
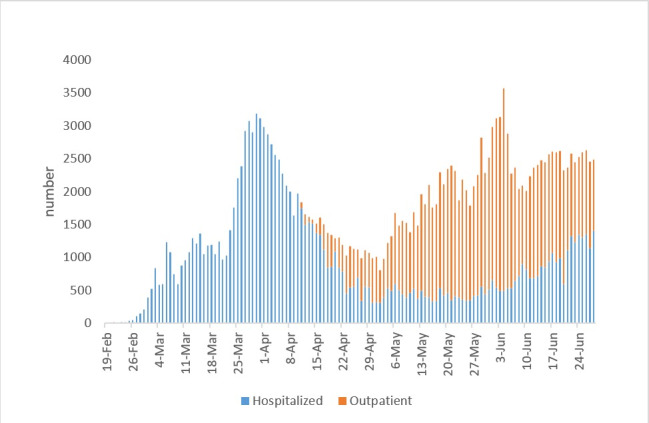
Daily reported of COVID-19 cases in Iran based on hospitalized and outpatients cases since 19 February to 26 June 2020

The number of cases showed a surge on 4 June 2020 (n=3574). This observation suggests that Iran may have experienced its second wave of the epidemic. Disaggregation of cases based on hospitalization status showed that much of the cases identified during the second wave were outpatients with mild symptoms (Outpatient/inpatient ratio≅ 4; Figure 1).

**Figure 2:**
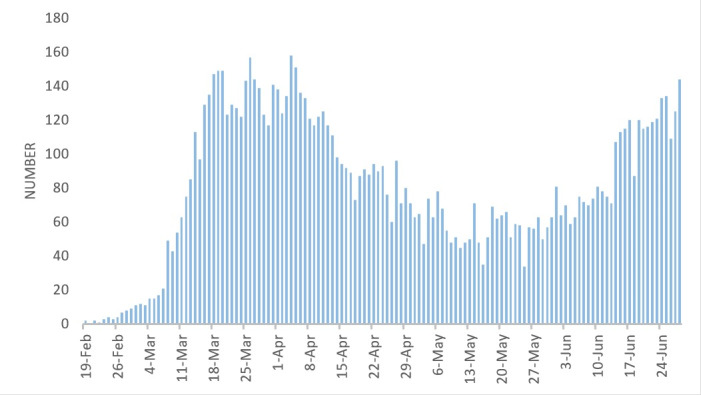
Daily reported deaths due to COVID-19 in Iran up to 26 June 2020

An investigation of the death trend also shows a peak in early April as well as a downward trend in late April. During May, the number of death cases showed a stable trend with a daily number of deaths ranging between 50 and 75 cases. Then the number of deaths gradually increased until it reached to more than 100 daily deaths by mid-June (Figure 2).

## Discussion

Iran is ranked as the 17th and 18th country based on its area and population size, respectively. This country has 31 provinces, more than 1,300 cities, and more than 6,000 villages, with a great diversity in climate, culture, and customs, which may affect the epidemic curve of COVID-19.

The decrease in the number of cases, which last until the end of April, maybe due to extensive lockdowns of businesses, the extensive engagement of the community, and adherence to the social and physical distancing measures during the second half of March.

Non-compliance with infection prevention and control precautions in local levels can affect the national trends of the disease that may lead to controversial observations at the national level. The provinces of Iran have sometimes experienced different epidemic trends since the start of the epidemic, which together has shaped the national curve in Iran^4^. Province-level variations in the shape of the epidemic curve might be due to various factors such as the time of the virus introduction to the province, the rate of virus transmission, behavior of the residents in each province, and the climatic. Although we have had a national policy for testing and case finding, there were heterogeneities between provinces in the implementation of such policy and this might be a contributing factor in province-level variations in the shape of the epidemic curve. Analysis of the epidemic curve of provinces and megacities would be useful in providing a better interpretation of the epidemic curve in Iran^5,6^.

The increase in daily reported cases since early May can have three main reasons.

**Figure 3:**
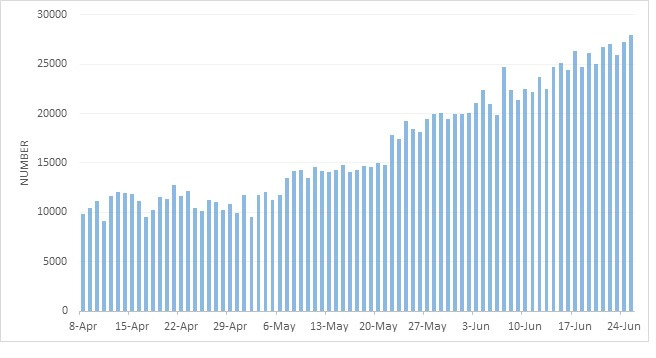
frequency of daily diagnostic testing of COVID-19 from 8 April to 26 June. (As 7 April, the number of cumulative COVID-19 tests was 211,136)

First, improvement in case-finding and high testing, which led to the increase in the number of daily cases from about 10,000 in early April to about 25,000 in the second week of June (Figure 3). In Iran, like most countries, the official daily reports of the disease are based on the confirmed laboratory cases. Since there was a limitation on PCR testing in Iran in the early weeks, as most other countries, the priority was to perform tests on hospitalized patients. From 11 April 2020, and after developing the country's testing capacity, the testing was scaled up to the community setting, which affected the epidemic curves of Iran. If the ability of the health system in testing was more during February to May 2020, it was expected that the first peek of the epidemic had more cases than what was observed, and its time was getting a little ahead or behind. With the assumption of no change in the severity of the disease, we expect to see the same proportion of outpatients and inpatients over the first wave of the epidemic as well. Therefore, it is anticipated to see no change in the general pattern of the first and second waves, and it is estimated to observe two separate waves still unless the first wave had been much larger than the official’s report.

Second, reducing the social and physical distancing measures by the community as well as the reopening of many businesses could have an important role in experiencing a second wave. Iran has experienced the epidemic before many other countries. The country also does not have a stringent policy for social distancing such as China. The reopening of businesses in Iran began on 9 April 2020, quite earlier than many other countries. Due to reopening of many businesses, non-compliance with the necessary considerations by the people and normalization of the situation, there was an increase again in the reported cases and death of the provinces that were affected by the epidemic in the first months, and it seems that the problem can come back.

Third, flare-up of the epidemic in some previously less-affected provinces in the south and west of Iran affected the overall epidemic curve by increasing the number of hospitalized and death cases. These provinces included Khuzestan, Hormozgan, Kermanshah, Kurdistan, Lorestan, East Azerbaijan, and West Azerbaijan with an overall population of about 19 million people. In any case that people do not comply with the social and physical distancing, more peaks of the outbreak are expected. As the upward trend can be a warning for starting a new wave, special attention is needed to timely implement effective control measures^5,6^.

One of the limitations of this study, like other studies analyzing the trend of the epidemics, is that the analysis is based on the identified and reported cases, while the quality of identification and reporting of cases may have changed over time, which may affect the real trend of the disease. Another issue with the interpretation of long-duration epidemics in all countries is changing the policy for screening and reporting the cases.

In the interpretation of the trend of the epidemic curve in Iran, even though changes in the capacity of daily testing, and variations in trends of the disease in different parts of the country should be taken into account, but it seems that a part of the increase in the number of cases and death is due to reopening of businesses and reducing the social and physical distancing by people and reduced epidemic control measures that need the attention of the people and the officials.

## Conclusion

It seems that the epidemic curve of COVID-19 in Iran is not comparable to many countries in the world. As the epidemic in Iran began earlier than many other countries in the world, and even though the pattern in other countries is most likely to be affected by their mitigation and suppression policy, it is predictable that some of the countries with similar situations experience the same pattern with a few weeks delay. The epidemic curve in Iran is a function of different factors such as number of total tests, change in mitigation policies, and heterogeneities among different provinces in the country and therefore it should be interpreted under the light of the effect of such factors.

## Conflict of interest

Dr. Ali Akbar Haghdoost is the Deputy Minister of Health and Medical Education in Iran. The rest of the authors have no conflict of interest to disclose.

## Funding

None.

## Highlights

- Epidemic curve in Iran is a function of total tests, changes in policies, and heterogeneities among provinces.
- Improvement in case-finding and high testing led to an increase in daily cases.
- Reducing the social and physical distancing had an important role in the second wave.
